# Thinking Outside the Frame: Impacting Genomes Capacity by Programmed Ribosomal Frameshifting

**DOI:** 10.3389/fmolb.2022.842261

**Published:** 2022-02-14

**Authors:** Ricarda J. Riegger, Neva Caliskan

**Affiliations:** ^1^ Helmholtz Centre for Infection Research (HZI), Helmholtz Institute for RNA-Based Infection Research (HIRI), Würzburg, Germany; ^2^ Graduate School of Life Sciences (GSLS), University of Würzburg, Würzburg, Germany; ^3^ Medical Faculty, University of Würzburg, Würzburg, Germany

**Keywords:** RNA, viruses, frameshifting, ribosome, translational regulation, translation

## Abstract

Translation facilitates the transfer of the genetic information stored in the genome via messenger RNAs to a functional protein and is therefore one of the most fundamental cellular processes. Programmed ribosomal frameshifting is a ubiquitous alternative translation event that is extensively used by viruses to regulate gene expression from overlapping open reading frames in a controlled manner. Recent technical advances in the translation field enabled the identification of precise mechanisms as to how and when ribosomes change the reading frame on mRNAs containing *cis*-acting signals. Several studies began also to illustrate that *trans*-acting RNA modulators can adjust the timing and efficiency of frameshifting illuminating that frameshifting can be a dynamically regulated process in cells. Here, we intend to summarize these new findings and emphasize how it fits in our current understanding of PRF mechanisms as previously described.

## Introduction

**FIGURE 1:**
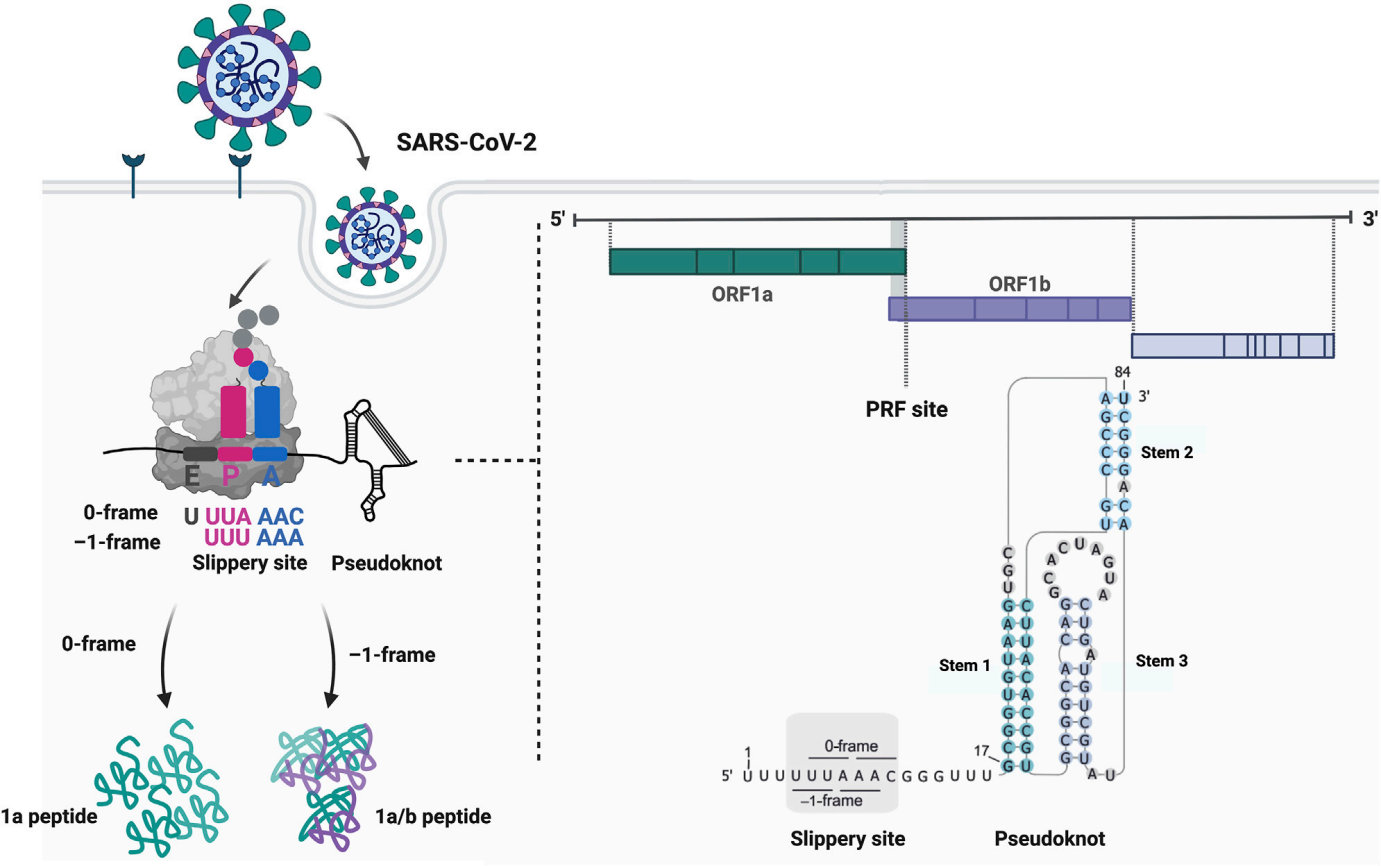
Programmed ribosomal frameshifting on the SARS-CoV-2 mRNA. Frameshifting on the Severe Acute Respiratory Syndrome Coronavirus 2 (SARS-CoV-2) mRNA occurs on the slippery sequence located at the overlap of the open reading frames 1a (ORF1a) and 1b (ORF1b). Here, the slippery sequence has the motif U_UUA_AAC followed by a short spacer and the frameshift stimulatory pseudoknot. This secondary structure element comprises a kinetic roadblock that precisely stalls the ribosome on the slippery sequence potentially leading to the movement of the ribosome by one nucleotide into the 5′ direction. The translocation continues in the –1-frame with the peptidyl P- and aminoacyl A-site codons UUU_AAA resulting in the synthesis of the 1a/b peptide. Created with BioRender.com.

Protein synthesis is essential for any living cell. Based on the genetic information encoded in the messenger RNA (mRNA) sequence, the ribosome catalyzes the peptide bond formation of each amino acid to the nascent polypeptide chain. The incorporation of the correct amino acid is facilitated by the match between the mRNA codon and its cognate anticodon of the tRNA that delivers the appropriate amino acid. The genetic code is universal and read in triplets directed from the mRNA 5′ to 3′ end. The movement of tRNAs and the mRNA through the ribosome is maintained by coordinated, inter- and intra-subunit conformational changes and rotations of the ribosome (Rodnina and Wintermeyer, 2011; Achenbach and Nierhaus, 2015; Noller et al., 2017; Rodnina, 2018). During canonical translation, the elongation process is synchronized with translocation of the ribosome by exactly one codon after resolving intra-molecular base-pairs by the ribosomal mRNA helicase located at the mRNA tunnel entrance (Takyar et al., 2005). Errors in the maintenance of the correct reading frame, referred to as spontaneous frameshifting, occur less than 10^−5^ times per codon during translation (Kurland, 1992). An interesting feature of many genomes is that they contain overlapping open reading frames (ORF) (Veeramachaneni et al., 2004; Firth, 2014; Pavesi et al., 2018; Schlub and Holmes, 2020) some of which can be accessed during translation via recoding (Jacks and Varmus, 1985; Brierley et al., 1987; Jacks et al., 1988a). Translational recoding events are employed to fine-tune gene expression and expand the genomic coding capacity. Unlike erroneous translation, these translational recoding sites contain specific features embedded in the mRNA to signal the ribosome to move to the alternative ORF in a programmed manner. Several forms of recoding exist: 1) programmed ribosomal frameshifting (PRF); 2) translational bypassing or leaky scanning of the first start codon by the 48S pre-initiation complex; and 3) stop-codon readthrough (Atkins and Gesteland, 2010; Brierley et al., 2010; Caliskan et al., 2015; Miras et al., 2017; Dinman, 2019a; Rodnina et al., 2020). While there are numerous reviews which extensively detail general mechanisms and occurrences of recoding events, namely (Gesteland and Atkins, 1996; Ketteler, 2012; Caliskan et al., 2015; Atkins et al., 2016; Dever et al., 2018; Dinman, 2019b; Rodnina et al., 2020), in this review we focus mainly on PRF, where a different reading frame is accessed through controlled slippage of the ribosome on an mRNA (Figure 1).

Cases of PRF have been reported in many viruses and domains of life such as on the bacterial *Escherichia coli dnaX* gene (Tsuchihashi and Kornberg, 1990), in archaea like in *Sulfolobus solfataricus* on the *α-l-fucosidase fucA1* mRNA (Cobucci-Ponzano et al., 2006), as well as in eukaryotes on the human embryonic *Paternally Expressed Gene 10* (*PEG-10*) (Manktelow et al., 2005; Clark et al., 2007). Movement of ribosomes during PRF can occur in both the + or − direction relative to the 5′ end of the mRNA by one to even six nucleotides (Weiss et al., 1987; Lainé et al., 2008; Fang et al., 2012; Yan et al., 2015). –1PRF, where the ribosome slips by one nucleotide in the 5′ direction is the best-known variety of PRF, but +1PRF (slippage by one nucleotide in the 3′ direction), e.g., first discovered on a transposon element in yeast (Ty) (Clare and Farabaugh, 1985; Clare et al., 1988; Belcourt and Farabaugh, 1990) and –2PRF in case of the Porcine Reproductive and Respiratory Syndrome Virus (PRRSV) (Fang et al., 2012) have been reported as well.

–1PRF is extensively studied in RNA viruses, including the coronaviruses [e.g., Infectious Bronchitis Virus (IBV), Severe Acute Respiratory Syndrome Coronavirus (SARS-CoV)] (Figure 1) and retroviruses [e.g., Human Immunodeficiency Virus (HIV)]. While the 5′ end of viral frameshift genomes usually encodes for structural proteins, the 3′ alternative ORF mostly encodes for proteins involved in replication and processing (Atkins et al., 2016). Therefore, PRF events represent an elegant way to regulate packaging and replication of viral genomes. The ratio of the upstream and the downstream, alternative translation products is referred to as frameshifting efficiency. Perturbations in frameshifting levels can alter viral spread and pathogenesis (Brierley et al., 1991; Dinman and Wickner, 1992; Hung et al., 1998; Shehu-Xhilaga et al., 2001; Plant et al., 2005; Dulude et al., 2006). The frameshifting efficiency varies widely from only 1% of all translation events on the Barley Yellow Dwarf Virus (BYDV) genome (Barry and Miller, 2002), to up to 80% in Theiler’s Murine Encephalomyelitis Virus (TMEV) (Finch et al., 2015). In yeast, predicted -1PRFs mostly would result in the termination of protein synthesis at premature termination codons in the alternative frame (Jacobs et al., 2007), which was shown to also trigger nonsense-mediated mRNA decay (NMD) and no-go decay (NGD) in order to clear the cells from non-functional mRNAs (Belew et al., 2011). Furthermore, when ribosomes stall or collide on frameshift sites, NGD is also commanded to dissolve stalled or collided elongation complexes by degrading the mRNA (Simms et al., 2019; Smith et al., 2019). Therefore, in addition to its role in expanding the genomes repertoire, frameshifting events allow adaptation of the encoded proteome to changes in cellular and environmental conditions or infections (Rom and Kahana, 1994; Matsufuji et al., 1995; Baranov et al., 2002; Caliskan et al., 2017; Meydan et al., 2017; Korniy et al., 2019).

## *Cis*-Acting Elements are Crucial for –1PRF

In most cases, the propensity of a ribosome to undergo –1PRF depends on two crucial *cis*-acting elements in the mRNA that are separated by a spacer sequence: a heptanucleotide slippery sequence on which the ribosome can slip into the alternative frame (Jacks et al., 1988a) and a downstream secondary structure element that causes the ribosome to slow down on the slippery sequence (Brierley et al., 1989).

The canonical slippery sequence is mostly a heptanucleotide motif that allows codon and anticodon base-pairing in both, the 0 and the alternative –1 reading frames (Jacks et al., 1988a; Jacks et al., 1988b; Icho and Wickner, 1989; Horsfield et al., 1995) (Figure 1). The most common slippery motif is X_XXY_YYZ (0-frame), where X can be any nucleotide, Y either adenine or uridine and Z any nucleotide except for guanine (Jacks et al., 1988a; Jacks et al., 1988b; Brierley et al., 1989; Dinman et al., 1991; Brierley et al., 1992), however also divergent patterns have been reported (Firth and Atkins, 2009; Loughran et al., 2011). Prior to slippage, the ribosome P- and the A-sites occupy the XXY and YYZ codons (0-frame) and during frameshifting, the ribosome moves to the XXX and YYY codons (–1-frame), which may lead to a mismatch of the anticodon and the codon in the wobble position. The spacer (five to nine nucleotides in eukaryotes, five to six nucleotides in prokaryotes) separating the slippery sequence and the secondary structure element ensures correct positioning of the ribosome on the slippery sites (Kontos et al., 2001; Howard et al., 2004; Lin et al., 2012; Napthine et al., 2017). The secondary structure element constitutes a kinetic barrier that slows down or stalls the translating ribosome (Brierley et al., 2010; Caliskan et al., 2014; Kim et al., 2014). This *cis*-acting element varies from a simple stem-loop [e.g., in the HIV-1 mRNA (Jacks et al., 1988a)], to a more complex H-type pseudoknot [e.g., in coronaviral mRNAs (Brierley et al., 1989)]. In exceptional cases, guanine-rich sequences that form four-stranded G-tracts referred to as G-quadruplexes, can form a physical barrier that is capable of stalling the ribosome similar to the stem-loops or pseudoknots reflecting the structural diversity of stimulatory RNA structures (Endoh et al., 2013; Yu et al., 2014). Of note, frameshift RNAs likely exist in several conformations with different stimulatory potentials (Houck-Loomis et al., 2011; Halma et al., 2019; Schlick et al., 2021). Also, the high structural variation of different stimulatory elements suggests that there could be mechanistic differences in how they act on the ribosome during translation elongation (Dinman, 1995; Kontos et al., 2001; Plant and Dinman, 2005). For instance, forming a translational roadblock is not the only way structured RNAs can alter recoding (Plant and Dinman, 2005). Frameshift RNA elements can also sterically obstruct the tRNA binding: Cryo-EM studies of the HIV-1 frameshift site have recently proposed this –1PRF activating function by revealing that the stimulatory HIV-1 stem-loop sterically hinders the binding of an aminoacylated tRNA to the A-site of the bacterial ribosome (Bao et al., 2020; Bao et al., 2021).

In addition to the slippery sequence and the downstream RNA structures, the presence of additional upstream RNA elements, such as Shine-Dalgarno-like sequences interacting with the 16S ribosomal RNA in *E. coli* (Larsen et al., 1994; Choi et al., 2020) or the frameshift attenuator sequence found upstream of the SARS-CoV frameshift site (Su et al., 2005; Kelly et al., 2020) were shown to modulate the levels of PRF. Another interesting feature of the SARS-CoV mRNA is that it does not only regulate frameshifting via its secondary structure in *cis*, but also in *trans* by forming dimers through kissing loop-loop interactions involving the stem 3 of the genomic RNA (Ishimaru et al., 2013; Bao et al., 2020; Bao et al., 2021). Such kissing loop interactions likely compete with the folding of the pseudoknot structure and thereby reduce the level of frameshifting. Overall, these illuminate that the regulatory *cis*-elements can vary in their structural folds and functions and how they work together with the canonical *cis*-acting stimulators of frameshifting remains to be studied.

## *Trans*-Acting Regulators of –1PRF Efficiency

Although classically, PRF was thought to depend on *cis*-acting RNA elements, similar to other RNA-based regulatory events, frameshifting levels can be modulated by *trans*-acting factors in cells. These *trans*-factors are pathogen- or host-encoded proteins or other molecules that either directly bind to specific mRNA motifs or the ribosome, or indirectly affect translation by interacting with other proteins (Penn et al., 2020). Such interactions would likely alter the thermodynamic stability of the stimulatory structure or impair kinetics of ribosomal translocation resulting in changed recoding rates.

Earliest examples of frameshifting regulation were reported to occur on the +1 frameshift mRNAs, human ornithine decarboxylase antizyme (Rom and Kahana, 1994; Matsufuji et al., 1995) and the *E. coli* release factor 2 (RF2) (Baranov et al., 2002), where the levels of polyamines and RF2 in cell have autoregulatory functions.

Protein-mediated –1 frameshifting has been more recently discovered on the cellular poly-(C) binding proteins (PCBP) that promote –1 as well as –2 frameshifting in arteriviruses such as the PRRSV by directly interacting with the viral nonstructural protein nsp1β (Napthine et al., 2016; Li et al., 2019). In addition to stimulation of –2 frameshifting, here also the lack of a typical downstream RNA structure is remarkable. Instead, frameshifting is mediated through the binding of the PCBP and the viral nsp1β to a cytosine-rich sequence (CCCANCUCC) on the mRNA downstream to the slippery sequence, which mimics a stimulatory secondary structure (Napthine et al., 2016; Li et al., 2019).

Another elegant example of a pathogen-encoded *trans*-factor-mediated translational regulation has been discovered in the cardioviruses Encephalomyocarditis Virus (EMCV) and Theiler’s Murine Encephalomyelitis Virus (TMEV) (Loughran et al., 2011; Hill et al., 2021b). The coding region of cardioviruses contain a conserved frameshift site at the 2A-2B ORF junction. Frameshifting occurs on the slippery motif G_GUU_UX eleven to twelve codons from the start of the 2B gene, producing a shorter 2B* protein, with undefined functions (Loughran et al., 2011; Napthine et al., 2017). However, it is assumed that the main function of this frameshift is the downregulation of the downstream encoded proteins, which in a way acts like a regulator of viral replication and assembly. Similar to PRRSV (Li et al., 2014), the downstream RNA motif, in this case a 35-nucleotide long stem loop, downstream of the slippery sequence is not sufficient alone to stimulate frameshifting. Frameshifting depends on an RNA-protein complex formed between the stem loop and the 2A protein. Cardioviral 2A is regarded as a multifunctional protein with key functions in virulence (Caliskan and Hill, 2022). In addition to regulating apoptosis, 2A was previously shown to bind to ribosomes and regulate translation (Groppo and Palmenberg, 2007; Hill et al., 2021b). Furthermore, it was discovered that 2A expression increases over the course of EMCV infection correlating with an increasing frameshifting efficiency of up to 70% (Napthine et al., 2017). The increase in 2A levels thus shuts down translation of downstream lying genes in the 0-frame, thereby ensuring appropriate levels of viral replicative proteins at early versus late stages of infection.

**FIGURE 2:**
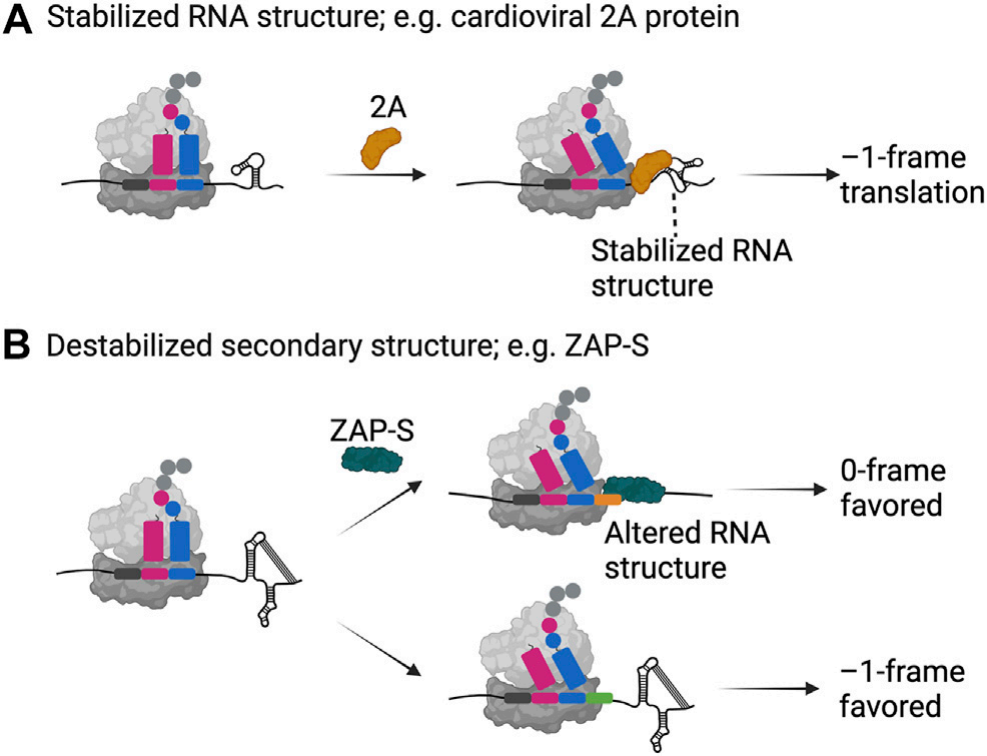
Examples of *trans*-factor-mediated frameshifting. Two examples, namely **(A)** the cardioviral 2A protein and **(B)** the zinc-finger antiviral protein ZAP-S are shown as representatives of how host- and pathogen-encoded proteins can alter the secondary structure of a frameshift mRNA resulting in enhanced or decreased frameshifting efficiencies. Both proteins specifically bind to the appropriate mRNA and interact with the ribosome. While the 2A protein binds and stabilizes the secondary structure of the EMCV mRNA leading to –1 frameshifting, ZAP-S binding contrastingly destabilizes the secondary structure element on the SARS-CoV-2 mRNA resulting in significantly decreased frameshifting efficiencies. Created with BioRender.com.

Recent work combining structural, biochemical and single-molecule analysis illuminated how the unique RNA binding fold found in the 2A structure allows it to interact with translating ribosomes and the downstream RNA element (Hill et al., 2021a; Hill et al., 2021b) (Figure 2A). The 2A protein interacts with the RNA at high affinity in a 1:1 stoichiometry (Hill et al., 2021a; Hill et al., 2021b). Furthermore, it also interacts with empty and translating ribosomes in a 3:1 stoichiometry possibly interfering with the binding of elongation factors on the ribosome. Detailed single-molecule analysis of the RNA-protein interactions of the wild type and mutant RNAs explained that 2A binding indeed stabilizes the EMCV RNA structure to a level that it alters the speed of translation elongation (Hill et al., 2021b) (Figure 2A). Combination of the higher force needed to unfold the structure and interference in binding of elongation factors caused by cardioviral 2A protein binding may thus explain the increase in the frameshifting efficiency (Groppo and Palmenberg, 2007; Loughran et al., 2011; Napthine et al., 2017; Hill et al., 2021b).

Such viral-encoded factors seem to selectively recognize particular RNA motifs and stabilize the RNA to impede translational elongation. Pausing at these sites likely opens a favorable time-window for codon-anticodon interactions to be re-established in the alternative reading frame, however, exactly which step of translation elongation is affected is still an open question.

The aforementioned findings also bring up the question whether modulation of frameshifting is a common feature of other cellular RNA-binding proteins as well. In this context, an earlier study by Kwak et al. showed that human annexin A2 (ANXA2) protein is associated with the IBV frameshift RNA element *in vitro* (Kwak et al., 2011). Interestingly, the knockdown of this factor increases frameshifting levels, suggesting a different mode-of-action than viral regulators of frameshifting. However, since ANXA2 is one of the most abundant proteins in the human cytoskeleton, it remains unclear how selective and conserved the interaction of ANXA2 and the IBV mRNA is or how the factor actually modulates frameshifting.

Other host factors also seem to recognize frameshifting ribosomes including the eukaryotic release factor 1 (eRF1) (Kobayashi et al., 2010) and the interferon-induced shiftless (SHFL) (Wang et al., 2019; Napthine et al., 2021; Zimmer et al., 2021). The cellular eRF1 was shown to interact with at least 30 other proteins in the cell together with HIV-1 proteins, therefore, what causes the decrease in HIV-1 frameshifting upon overexpression of the eRF1 remains open (Kobayashi et al., 2010). Another cellular protein SHFL, previously named RyDEN or C19orf66 is an interferon-induced protein which was reported to inhibit replication of some viruses like Dengue Virus (DENV) (Suzuki et al., 2016). Contrary to Dengue Virus which does not frameshift, HIV-1 mRNA frameshifting decreases through SHFL interaction suggesting multiple antiviral functions for SHFL (Wang et al., 2019). This results in altered stoichiometry of the structural Gag protein to the Gag-Pol polypeptide ultimately leading to inhibition of HIV-1 replication. It was suggested that SHFL recruits cellular release factors to stalled ribosomes (Wang et al., 2019), but how the factors recognize frameshifting versus other stalled ribosomes awaits investigation. Beyond HIV-1, SHFL was shown to act broadly on other viral and cellular recoding sites including SARS-CoV-2 (Napthine et al., 2021; Schmidt et al., 2021; Zimmer et al., 2021) and the cellular *PEG-10* mRNA (Wang et al., 2019). In summary, these studies suggested that cellular proteins work in a concerted way to interfere with viral RNA frameshifting regimes. However, whether this also occurs in an mRNA-specific manner like in the case of viral 2A protein has been unclear.

The short isoform of the cellular zinc-finger antiviral protein ZAP, ZAP-S was recently identified as a *trans*-acting factor directly influencing –1PRF during SARS-CoV-2 infections (Zimmer et al., 2021). This interferon-induced protein (Yang and Li, 2020) impairs –1PRF, which is pertinent for the synthesis of the viral RNA polymerase through its specific interactions with the frameshift RNA pseudoknot (Zimmer et al., 2021) (Figure 2B). Among other frameshift sites tested, the effect was only observed for SARS-CoV-1 and-2, which differ by only one nucleotide in the primary sequence of the putative structure, suggesting specific interactions of ZAP-S with the SARS-CoV frameshift RNA element and an alternative mode-of-action compared to the host factor SHFL. By *in vitro* ensemble and single-molecule analysis, Zimmer and Kibe et al. showed that ZAP-S preferentially binds to the stem-loops 2 and 3 of the pseudoknot. Accordingly, unlike the cardioviral 2A protein, human ZAP-S does not stabilize the viral frameshift RNA, instead it interferes with the folding of the pseudoknot (Figure 2B). The interaction of ZAP-S with the frameshift site thus alters the stability of the secondary structure, which then no longer constitutes a blockade for the translating ribosome. Reduced frameshifting rates would lead to a drop in the viral polymerase level and consequently impede viral replication (Zimmer et al., 2021). Overall, despite following different modulatory mechanisms, host factors seem to have a common inhibitory effect on ribosomal frameshifting on viral mRNAs, suggesting that cells developed this type of global or gene-specific strategies as part of the antiviral response.

Interestingly, not only proteins have been categorized as *trans*-factors influencing the frameshifting efficiency but also other molecules: Small molecules including small RNAs [e.g., locked nucleic acids (LNAs), micro RNAs (miRNAs)] have already been successfully used for *in vitro* studies on frameshifting and consequently be suggested to act as potential effectors of the frameshifting process (Plant and Dinman, 2005; Henderson et al., 2006; Yu et al., 2010; Matsumoto et al., 2018; Puah et al., 2018; Chen et al., 2020; Sun et al., 2021). Whether such molecules are stable and specific enough to allow a precise tuning of gene expression warrants further studies.

In conclusion, ultimately it can be assumed that many more *trans*-factors await discovery. Whether in the form of proteins or other molecules affecting the frameshifting process, regulation of PRF seems to be a layer of host-pathogen interaction which is only recently been recognized. Furthermore, despite the fact that numerous studies already contributed pieces to better understand this recoding event, the molecular mechanisms by which these modulators act on the ribosomal frameshifting routes need to be further elucidated.

## Other Factors that Alter Frameshifting

**FIGURE 3:**
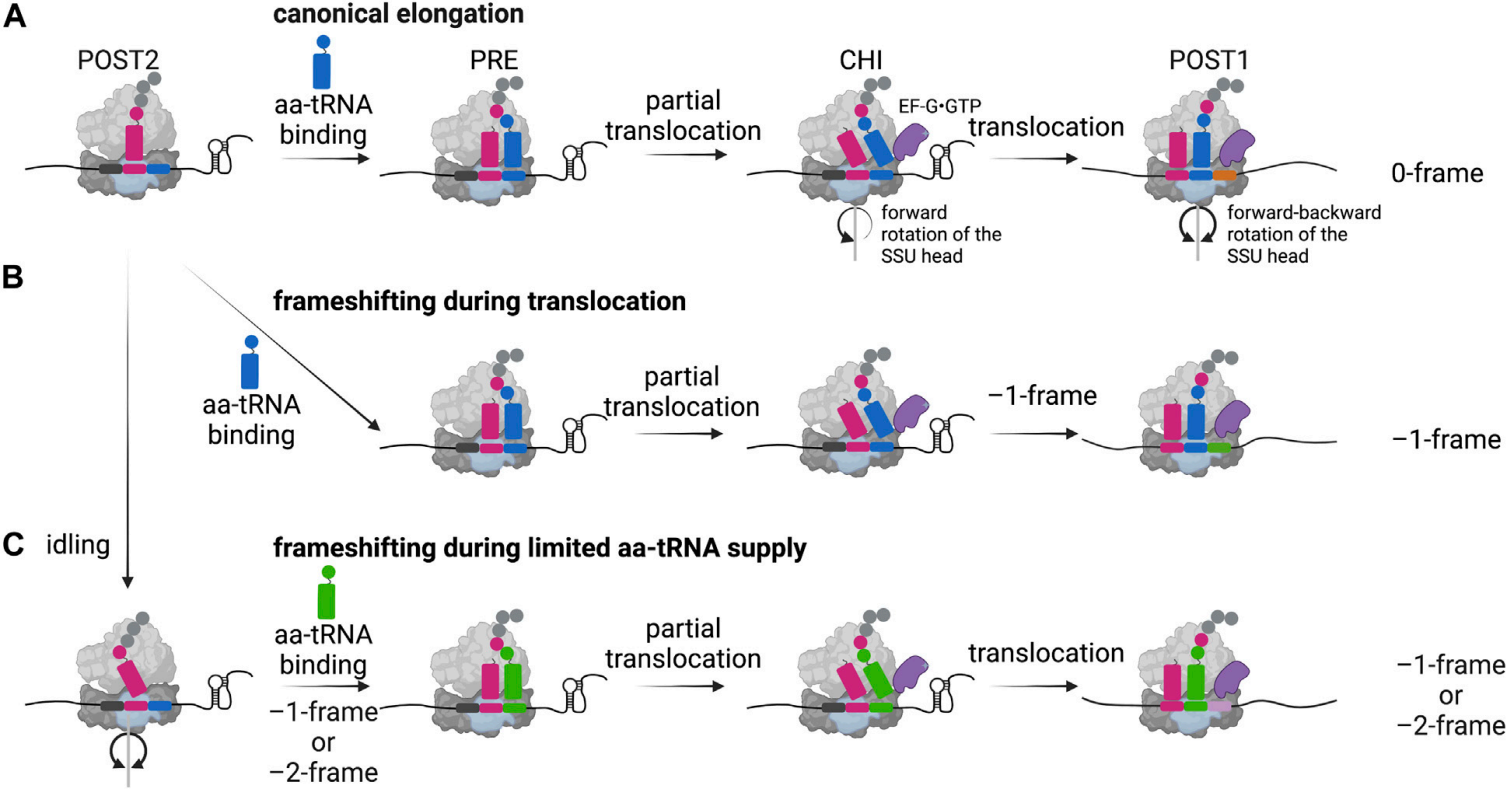
–1 Programmed ribosomal frameshifting models. **(A)** In the canonical elongation pathway, movement of the ribosome along the mRNA and the peptide bond formation is facilitated by specific forward and backward movements of the small subunit (SSU) head and the hydrolysis of GTP by the elongation factor EF-G (eEF-2 in eukaryotes). POST: post-translocation state, PRE: pre-translocation state, CHI: chimeric state. **(B)** In case of canonical –1 frameshifting, ribosomal stalling is caused by a secondary structure of the mRNA leading to frameshifting during translocation. **(C)** An alternative frameshifting pathway is mediated by the limitation of the aminoacyl-site codon respective aminoacylated tRNA (aa-tRNA), here an idling step gives the time to overcome the limitation by shifting into the –1- or –2-frame. Created with BioRender.com.

The efficiency of frameshifting can be modulated by a variety of other effectors that tune the overall fidelity and rate of translation. Among those, limitations of aminoacylated tRNA supply can lead to an alternative frameshifting pathway, also referred to as hungry-codon frameshifting (Clare and Farabaugh, 1985; Clare et al., 1988; Belcourt and Farabaugh, 1990; Barak et al., 1996; Caliskan et al., 2017) (Figure 3C). Here, the frameshifting occurs when the A-site of the ribosome is vacant since the aminoacylated tRNA substrate is limited. After the slippage of the tRNA bound in the P-site, decoding continues in the alternative reading frame. Furthermore, it was shown that this conditional frameshifting can occur even by two nucleotides in consecutive steps and does not depend on the stimulatory secondary structure element (Caliskan et al., 2017). In addition, not only the aminoacylated tRNA supply, but also the presence and degree of certain tRNA modifications influences frameshifting: Particularly ones in the anticodon loop or on position 37 of the tRNA can lead to weakened and inefficient base-stacking interactions between the codon and the anticodon and alter the frameshifting process (Bjork et al., 1989; Urbonavicius et al., 2001; Licznar et al., 2003; Tükenmez et al., 2015). Similar to −1 frameshifting, also +1 frameshifting levels can be affected when tRNAs contain mutations like insertions in the anticodon stem-loop or when modifications are missing (O’Mahony et al., 1989; Atkins and Bjork, 2009; Hoffer et al., 2020; Gamper et al., 2021b).

Interactions of the nascent polypeptide chain with the ribosomal exit tunnel can lead to translational stalling (Wilson and Beckmann, 2011). +1PRF efficiency was previously shown to be regulated by the interactions of the nascent peptide (Yordanova et al., 2015). Recently, stalling caused by co-translational folding of the polypeptide was reported to influence −1PRF (Harrington et al., 2020; Carmody et al., 2021). It was shown that co-translational folding during the integration of an isomer of the structural polypeptide of the alphavirus Sindbis Virus (SINV) into the endoplasmic reticulum (ER) membrane correlates with frameshifting efficiency (Harrington et al., 2020). Especially, mutations altering the composition of the nascent polypeptide, especially charged amino acids located immediate upstream of the slippery sequence could be crucial for this effect (Harrington et al., 2020; Carmody et al., 2021). Based on molecular dynamic simulations, the group suggested that the folding of the protein generates a mechanical tension on the nascent polypeptide chain which alters −1PRF rates (Carmody et al., 2021). More recently, the co-translational folding of the nascent polypeptide chain of the viral non-structural protein 10 (Nsp10) was determined to distinctly interact with the ribosomal tunnel resulting in an upregulated frameshifting level on SARS-CoV-2 mRNA (Bhatt et al., 2021). Furthermore, mutations within the ribosome affecting their translation accuracy (Brunelle et al., 1999), mutations in the 16S ribosomal RNA platform region of the small ribosomal subunit (Leger et al., 2007) as well as mutations in proximity to the ribosomal exit E-site (Robert and Brakier-Gingras, 2003) were shown to alter the reading frame. Dependent on the ribosome loading on a given mRNA, frameshifting events may be altered and regulated by trailing ribosomes (Smith et al., 2019). Evidence has also been presented for the possibility that loading of the ribosome and rate of initiation on an mRNA inversely correlates with the formation of the stimulatory structure and thus alters frameshifting (Gendron et al., 2008).

In addition, components of the translational machinery can affect the overall fidelity of translation and therefore are important for the maintenance of the reading frame. Especially the elongation factors eEF1A and eEF2 in eukaryotes and their counterparts EF-Tu and EF-G in prokaryotes are crucial: Through their GTPase activities, elongation factors deliver the essential energy for binding of the aminoacylated tRNA to the A-site and the ribosomal translocation along the mRNA. Mutants of EF-Tu or eEF1A were shown to promote frameshifting (Dinman and Kinzy, 1997). Also, the eukaryotic translocation elongation factor 2 (eEF2) driving tRNA translocation was highlighted to be important for maintaining the reading frame since mutations in only one amino acid can affect translation fidelity leading to frameshifts (Ortiz et al., 2006). In particular, mutants of the domain IV of eEF2 in yeast (Ortiz et al., 2006; Peng et al., 2019) and mice (Liu et al., 2012) increased -1 frameshifting occurrence despite unaffected ribosome binding and GTP hydrolysis.

All in all, these suggest that the level of frameshifting is defined by multiple factors in the cell and support that the reading frame can be modulated in a time- and/or tissue-specific manner. Furthermore, the aforementioned examples show that, when ribosomal frameshifting is investigated, light should be also shed on the ribosomal exit tunnel to consider the influence that the interaction of the nascent polypeptide chain and the tunnel has on regulating frameshifting. The complex regulatory network also raises the question, what the molecular determinants are that cause the ribosome to slip into the alternative reading frame on a given coding sequence?

## Molecular Determinants of Ribosome Pausing at Secondary Structures

A fundamental understanding of how various *cis*-acting elements and *trans*-acting factors act in a concerted way during the transition from the regular to the alternative translation route requires a detailed kinetic framework of the molecular events defining PRF. Current knowledge points to the existence of several determinants of how RNA elements can mediate pausing and lead to frameshifting. These include the thermodynamic stability of the stimulatory elements as well as the conformational heterogeneity and structural plasticity of the stimulatory secondary structure. Regardless of how the kinetic barrier is formed, it is commonly accepted that such elements should sufficiently slow down or stall the ribosome, thereby increase the time-window that the tRNA dwells on the slippery codons (Caliskan et al., 2014; Chen et al., 2014; Kim et al., 2014; Choi et al., 2020).

Thermodynamic stability and the unfolding kinetics of the frameshift stimulatory structures are crucial determinants of the translational pause (Plant and Dinman, 2005; Chen et al., 2009; Giedroc and Cornish, 2009; Mouzakis et al., 2013; Caliskan et al., 2014; Choi et al., 2020). Evidence comes from earlier findings reporting that the local thermodynamic stability of the bottom part of the stem in the *dnaX* frameshift mRNA is more stable than the upper part (Larsen et al., 1997) and the stability of the first three to four base-pairs of the HIV-1 frameshift-mediating stem-loop positively correlates with frameshifting efficiency (Mouzakis et al., 2013). Here, the replacement with base-pairs of slightly higher stability results in an enhanced frameshifting efficiency. Studies comparing various stimulatory elements demonstrated that the local stability of the mRNA secondary structure is important for pausing and energetic restraints during ribosome movement due to the high energy needed for unwinding of the supercoiled stem is crucial for the frameshifting process (Plant and Dinman, 2005; Mouzakis et al., 2013; Caliskan et al., 2014). In addition, Choi et al. proposed a correlation between the duration of ribosome pausing and the number of base-pairs that has to be resolved before the ribosome fully translocates (Choi et al., 2020). Moreover, the precise length of the spacer region between the slippery codon and the frameshift stimulatory element on the mRNA ensures the correct positioning of the ribosome at the P-site codon during frameshifting (Kontos et al., 2001; Howard et al., 2004), which seem to place the base of the pseudoknot at the active site of the ribosomal helicase (Takyar et al., 2005; Caliskan et al., 2014). Consistently, kinetic studies of ribosomes stalled on the IBV frameshift mRNA showed a four-fold faster translocation in case the ribosome slipped into the –1-frame compared to continued translation in the 0-frame (Caliskan et al., 2014). This confirms that the helicase activity associated with the ribosomal mRNA entry channel highly depends on its precise positioning relative to the secondary structure and changing the position by a single nucleotide into the –1-frame assists the ribosome to better unwind the highly structured stimulatory RNAs (Qu et al., 2011; Caliskan et al., 2014; Choi et al., 2020).

Two recent studies provided the mechanistic insights into the interactions of the frameshift RNA structures and the ribosomal helicase. Bao et al. observed an inhibitory interaction of the secondary structures on the *E. coli dnaX* as well as the HIV-1 mRNA with ribosomes, that could also interfere with A-site binding (Bao et al., 2020). Using ribosomes primed at the SARS-CoV-2 frameshift site, Bhatt and Scaiola et al. illustrated that the pausing prior to the frameshift and that specific interactions of ribosomal helicase proteins and the helix h16 of the 18S ribosomal RNA (rRNA) and the bases within the pseudoknot were important for frameshifting. A particular position on the stem loop 1, the guanine at position 13,486, was shown to flip out from its stacked position within the loop and interact with the ribosomal helicase protein uS3 (Bhatt et al., 2021). The authors also underlined an important role of this guanine residue of the pseudoknot by showing an up to 45% reduction in frameshifting rates when this base was mutated (Bhatt et al., 2021). Overall, these studies reveal mechanistic and regulatory features that influence the pause duration at the frameshift sites.

## Frameshift mRNAs can Exist in Several Conformations

Others also proposed that structural plasticity and conformational heterogeneity of the secondary structure strongly correlates with translational pause and frameshifting efficiencies (Ritchie et al., 2012; Wu et al., 2018; Dinman, 2019a; Halma et al., 2019; Neupane et al., 2021). Wu et al. proposed that the ribosomal helicase unwinds only single base-pairs at once but not the entire structure, leaving time for the secondary structure to fold into other intermediates (Wu et al., 2018). These intermediates may enhance the frameshifting efficiency since translation might be faster when continuing in the –1-frame than overcoming the higher energy barrier to unfold the new, more stable structure (Wu et al., 2018). Furthermore, different mRNA secondary structures divergent from the initial structure might be formed due to different orders in refolding during or after the ribosome moves over the sequence, resulting in an altered frameshifting rate after the initial round of translation (Lyon et al., 2019; Halma et al., 2021; Hsu et al., 2021; Neupane et al., 2021). Moreover, considering the aforementioned importance of the spacer length, frameshifting might also be affected by secondary structures including parts of the spacer sequence since the effective length between the two frameshift stimulatory sequences would be altered in this case (Napthine et al., 2017; Hill et al., 2021b; Omar et al., 2021). Ultimately, the conformational heterogeneity of the frameshift-stimulating structure has been revealed to correlate with the frameshifting efficiency (Halma et al., 2021; Neupane et al., 2021; Schlick et al., 2021). Nevertheless, these conclusions are based on diverse mRNA structures and differences between *in vivo* and *in vitro* studies should be taken into account.

## Thermodynamic Model of Frameshifting

The thermodynamic stability and the structural heterogeneity of the downstream RNA elements are important determinants defining the strength of the RNA roadblock and duration of the pause. However, propensity to frameshift on a given mRNA and the rate of frameshifting is primarily determined by thermodynamics of base-pairing between the individual bases of the codon and the anticodon of the slippery heptanucleotides. Early on, the interaction of the tRNA anticodon and the mRNA codon was highlighted to influence frameshifting (Tsuchihashi and Brown, 1992). Furthermore, the nature of the mRNA bases, wobble propensity of the tRNAs and the presence of certain modified and unmodified bases dictates how much frameshifting will take place (Licznar et al., 2003; Nguyen et al., 2019). Bock et al. confirmed that the frameshifting efficiency on a given mRNA is not defined by kinetic determinants of unfolding the secondary structure, but is mainly controlled by the free energy of base-pairing between the codon and the anticodon of the slippery sequence (Bock et al., 2019). Long pause of the ribosome at an mRNA secondary structure may serve to achieve sufficient time for the ribosome to explore the energetically more favorable state in the 0- or –1-frame to continue translation. Based on thermodynamic modelling of the energy landscapes of individual codon-anticodon interactions on the *dnaX* frameshift site, the authors illustrated that the frameshifting efficiencies on a given frameshift sequence can be predicted quantitatively (Bock et al., 2019).

In conclusion, these findings suggest that the thermodynamic constraints of the frameshift stimulatory elements, as well as the conformations might synergistically work to mediate stalling of translating ribosomes thus providing the essential time-window in which the ribosome can explore an alternative reading frame. However, ribosomes are thought to be already primed for frameshifting due to the thermodynamically favorable nature of base-pairing in the alternative reading frame.

## Timing of –1PRF During Translocation

Based on these molecular constraints, when exactly does ribosomal frameshifting happen during translation?

During protein synthesis, following the accommodation of the correct aminoacylated tRNA in the A-site and peptide bond formation, the resulting pre-translocation state (PRE) ribosome undergoes large conformational changes facilitating the translocation of the tRNAs from the P- and A-sites into the E- and P-sites, along the mRNA in one codon steps (Rodnina and Wintermeyer, 2011) (Figure 3A). Since ribosomes are highly dynamic during this process, the exact determination of when ribosomal frameshifting happens during translation requires observation of the elemental steps of the elongation cycle over frameshift motifs in real time. Several studies employed single-molecule and ensemble kinetic analysis techniques to directly follow the translation process shortly upstream, at and downstream of the frameshift site to explain the precise position of the ribosome during slippage as well as the timing of frameshifting (Caliskan et al., 2014; Chen et al., 2014; Kim et al., 2014; Yan et al., 2015; Caliskan et al., 2017; Kim and Tinoco, 2017). There is evidence from genetic mutational studies of elongation factors that reading frame can be altered in both directions during accommodation or translocation (Dinman and Kinzy, 1997; Ortiz et al., 2006; Liu et al., 2012; Peng et al., 2019). A computational model predicted that the kinetic parameters of aminoacylated tRNA binding, peptide bond formation and translocation define the simultaneous accessibility of –1PRF through different pathways (Liao et al., 2011). *In vitro* structural and biochemical studies suggested that translocation of the tRNAs is the step of elongation cycle when codon-anticodon interactions most likely are broken and reestablished in the new reading frame (Giedroc and Cornish, 2009). The codon resolved, stepwise kinetic analysis of –1PRF on the IBV frameshift site demonstrated that canonical slippage occurs at a late step of tRNA translocation, when the tRNAs both bound on the slippery codons move from the P- and A-sites into the E- and P-sites (Caliskan et al., 2014) (Figure 3B). Supporting these findings, frameshifting on the bacterial *dnaX* gene, which is stimulated by a stem loop, was shown to occur when the translocation of the two tRNAs bound to the slippery sequence codons is slowed down (Chen et al., 2014; Kim et al., 2014; Caliskan et al., 2017; Kim and Tinoco, 2017). Accordingly, the recruitment of EF-G to the PRE complex was shown to facilitate the tRNA movement into the chimeric (CHI) state, however, the presence of the secondary structure prevents the backward rotation of the small subunit head, which is essential for completion of translocation (Caliskan et al., 2014; Chen et al., 2014; Yan et al., 2015). This slow translocation results in delayed dissociation of the E-site tRNA and EF-G (Caliskan et al., 2014), in part also explaining an earlier reported eukaryotic frameshifting complex during pausing at the IBV pseudoknot with eEF2 bound to the ribosome (Namy et al., 2006). Biochemical studies also suggest that the ribosome would be trapped in an unusual chimeric state, which would undergo several futile attempts of incomplete translocation allowing the ribosome to explore alternative routes to resume translation (Caliskan et al., 2014; Kim et al., 2014; Yan et al., 2015; Kim and Tinoco, 2017; Desai et al., 2019). It was reported that the unfolding of the secondary structure only happens after EF-G binding and directly depends on the force generated when the small subunit head undergoes the forward rotation (Desai et al., 2019). In addition, the delay in the reverse rotation of the small ribosomal subunit head into the non-rotated state and thus the increased lifetime of the rotated state of the ribosome correlates with the frameshifting efficiency (Choi et al., 2020). Nevertheless, although simultaneous translocation seems to be the predominant pathway in these *in vitro* systems, other –1PRF routes can also operate, e.g., when aminoacylated tRNAs supply is limited (Clare and Farabaugh, 1985; Clare et al., 1988; Belcourt and Farabaugh, 1990; Barak et al., 1996; Caliskan et al., 2017) (Figure 3C). Furthermore, as mentioned earlier, mutations of the ribosome, translation factors and tRNA modifications can alter the kinetic parameters of frameshifting and favor alternative translocation pathways (Atkins et al., 2016). For instance, Chen et al. Reported that *dnaX* frameshifting can occur before accommodation of the second codon of the slippery sequence, but still when the ribosome is in the long paused hyper-rotated state (Chen et al., 2014).

Recent studies employing a +1 frameshift-prone mRNA and native *E. coli* tRNA (tRNA^Pro^) also suggested that timing of +1 frameshifting could be similar to canonical –1 frameshifting occurring during late steps of translocation (Demo et al., 2021). Structural studies of +1 frameshift complexes reported extensive conformational rearrangements of the 30S head and body domains mimicking what is observed in a translocation intermediate state interacting with EF-G (Hong et al., 2018). In this scenario, while the tRNA-mRNA base-pairing is dynamic during swiveling movement of the small subunit head, in frameshift-prone ribosomes EF-G fails to some extent to maintain the codon-anticodon interactions and allows slippage into the +1 reading frame (Demo et al., 2021). This points that both, +1 and –1 frameshifting can be driven by swiveling movement of the small subunit head domain (Gamper et al., 2021a; Demo et al., 2021). Nonetheless, in the absence of EF-G, +1 frameshifting can also be mediated by quadruplet interactions between the codon and the extended or modified anticodon loop of a single tRNA (Maehigashi et al., 2014). Whether the present models of frameshifting apply also to other organisms and details of frameshifting pathways in eukaryotes remain to be further investigated.

## Concluding Remarks

Despite fundamental knowledge gained about frameshifting events over the past years, there are still many outstanding questions concerning the detailed molecular mechanisms and occurrences of PRF. For instance, both upstream and downstream regions of frameshift RNAs seem to influence folding and the function of *cis*-acting canonical frameshift elements. Also, discovery of novel cellular and viral *trans*-acting factors, nascent polypeptide chain interactions and modifications on the RNAs affecting frameshifting, continuously reveal new levels of complexity. In the long run, the accumulated knowledge about this recoding mechanism and its regulation in cells will help to pave the way for therapeutic studies inhibiting frameshifting on pathogen mRNAs. Potential antiviral therapeutics could be designed to bind the frameshift RNA in a way that the stimulatory secondary structure either cannot be formed or cannot be unwound, hence prohibiting viral protein synthesis. In this respect, small synthetic, complementary oligonucleotides have already been suggested as possible therapeutic agents (Vickers and Ecker, 1992; Howard et al., 2004; Olsthoorn et al., 2004). Artificial, peptide nucleic acids (PNAs) can be designed to target secondary structures like hairpins and forming stable triplexes, thus enabling impacting –1PRF. Nevertheless, designing PNAs is challenging since the specificity and efficacy need to be high to prevent off-target effects. However, in potential cases where frameshifting might be supposed to be enhanced, the triplex needs to be resolved allowing the ribosome to continue translation in the –1-frame (Puah et al., 2018). Additionally, the discovery and optimization of novel small molecules that modulate frameshifting efficiencies such as the benzene derivative reagent RG501 (Hung et al., 1998), doxorubicin (Marcheschi et al., 2009), naphthyridine carbamate tetramer (Matsumoto et al., 2018) or merafloxacin (Sun et al., 2021) are desirable to find effective antiviral therapeutics. Collectively it is evident, that more research is imperative to fully understand the mechanism and all players involved in reinterpretation of the genetic code by frameshifting.
